# Crack Restraining Methods and their Effects on the Microstructures and Properties of Laser Cladded WC/Fe Coatings

**DOI:** 10.3390/ma11122541

**Published:** 2018-12-13

**Authors:** Qiu-Lian Dai, Can-bin Luo, Fang-yi You

**Affiliations:** College of Mechanical Engineering and Automation, Huaqiao University, Xiamen, Fujian 361021, China; lcbrobin@hqu.edu.cn (C.-b.L.); fangyiy@hqu.edu.cn (F.-y.Y.)

**Keywords:** Laser Cladding, WC/Fe coating, crack restraining methods, microstructures, mechanical properties

## Abstract

Laser cladded WC/Fe coatings have the advantages of low cost and high abrasion wear resistance. However, cracks always appear in WC/Fe coatings, which limits their industrial application. In this paper, the co-effects of the re-melting process, heat treatments, and amount of Co element on the cracking susceptibility, microstructures, and mechanical properties of WC/Fe laser cladding coatings were studied. Experimental results show that re-melting process is helpful to improve the surface quality of the coating and to reduce the cracking susceptibility. The hardness of the coating decreases slightly but distributes more uniformly. Cracks in the coating can be inhibited effectively by preheating the substrate to 250 °C and maintaining the temperature during the laser cladding process, as well as applying an annealing treatment at 300 °C for 1 h. Heat treatment also results in a slight decrease in the hardness. Crack initiation cannot be restrained completely by applying the above two methods when laser cladding a big area of coating. On the basis of the above two methods, addition of Co element to the coating can further improve its toughness and decrease the crack susceptibility. Crack-free WC/Fe coating can be manufactured when 8% Co is added, and its wear resistance is much better than that of the hardened medium steel, especially when the wear time is long.

## 1. Introduction

The development of hard-facing coatings has become technologically significant in many industries [1]. Laser cladding has benefits over conventional cladding (MIG, TIG, thermal spray) as laser cladding can produce dense coatings with low dilution and metallurgical bonding to the substrate [2,3]. Laser cladding with carbide ceramics particle-reinforced metal matrix composite coatings possess high hardness, high melting temperature, and good corrosion resistance. It is thus possible to easily produce a coating with new unique properties and promote it to be possible to modify properties of the surface of various materials such as light metals and various species of steels and cast iron [4,5,6]. Among the materials explored, tungsten carbide (WC)-reinforced Fe-based composite coatings are of considerable interest for their practical applications due to not only the abundant natural resources of Fe, but also the unique combination of the relatively low cost and high abrasion wear resistance [7]. However, the high cracking propensity of the coatings severely limits their applications. 

Laser cladding composite coatings are easy to generate crack due to the big temperature gradient induced by rapid heating/cooling during the laser cladding process and the widely different properties between the reinforcement and the base metal of the composite, as well as the different coefficient of expansion between coating and the substrate materials. Cracking susceptibility increases with the increase of the hardness and thickness of the coatings, especially for the reinforcement particles with high volume fraction because WC particles directly cause clad cracks by particle fracture under tensile stress [7,8,9]. 

Considerable research has been carried out to solve the cracking problem in laser cladding coatings [1,10,11,12,13,14]. It was reported that the cracking susceptibility can be decreased by reducing the thermal stresses in the coating via preheating and controlling cooling or by increasing the ductility of the coating through adding alloying elements to modify their phase constitution and microstructures. In addition, laser re-melting was considered as an effective method to improve the surface quality and properties of cladding coating [3]. W.L. Song et al. [10] studied the effects of Co on the cracking susceptibility of Fe–Cr–Ni laser clad layers. The results indicated that Co could effectively decrease the cracking susceptibility of the clad layer and at the same time Co does not change the hardness of the clad layer. The studies of W.Y. Gao et al. show that the addition of Ti into Fe-based alloy powder changes the morphology and microstructure of the coatings obviously. With the increase of Ti content, the dilution rate of the coatings goes up and the size of crystal phase in the deposits becomes larger, which makes the hardness of high-doped layers decrease sharply [13]. However, addition of Ta in a NiCrBSi alloy laser clad composite coating led to the in situ formation of fine TaC particles. Hence, the microhardness of the composite coating was improved and the crack susceptibility of the Ni based composite coating was reduced [14]. S.F. Zhou et al. [15] reported that preheating the substrate with a furnace or gas flame was an effective method to reduce the temperature gradient between the cladding layer and substrate, therefore preventing cracking. Y.F. Tao et al. [16] studied the residual stress distribution in different depths of TiNi/Ti_2_Ni-based laser clad coatings prepared at different environmental temperatures. Results showed that the average residual stress in the coating prepared at 25 °C was the largest. With the increase in environmental temperature, the average residual stress was reduced to zero when the environmental temperature was 800 °C.

Laser cladding composite coatings are mostly intended for wear applications. Hence, solving the cracking problem must not be accompanied by a significant loss of hardness. Though many studies about preheating the substrate and addition of alloy elements on the mechanical properties and crack susceptibility of the coatings have been carried out and have indicated they can toughen the coating and decrease its cracking propensity, the cracks of the coating cannot be completely eliminated, especially when laser cladding large pieces. In addition, studies on the combined effects of laser re-melting, preheating and annealing treatment, and Co addition on the microstructure, mechanical properties, and crack susceptibility of laser-clad WC/Fe composite coatings are limited.

In this study, the effects of the re-melting process, heat treatment, and amount of added Co on the cracking susceptibility, microstructure, and mechanical properties of WC/Fe laser cladding coating were studied. The final objective of this study is to explore the optimized heat treatment parameters and the amount of Co element to manufacture crack-free WC/Fe composite coatings without lowering their high hardness or deteriorating their wear resistance.

## 2. Materials and Methods

### 2.1. Materials

Laser cladding coatings of WC/Fe-based composite were fabricated on medium carbon steel substrates with 0.45% C (AISI 1045). Each substrate was abraded with Al_2_O_3_ abrasive papers of 46 mesh grain size and cleaned before being coated. Powders of Fe-based alloy, Co, and WC were mixed evenly in a MX-2 blender mixer and then be dried. The sizes of the Fe-based alloy power, Co, WC are 45–109 μm, 25–53 μm, and 10 μm respectively. Detailed chemical composition of the Fe-based alloy is presented in Table 1. The amount of WC in the coatings was set as 10% (weight percentage). Different amounts of Co were added to the Fe-based alloy.

### 2.2. Laser Clad Processing

The coatings with dimensions of 25 mm × 15 mm × 0.8 mm and 45 mm × 40 mm × 0.8 mm were made by preplacing the mixed powders on the substrate with the help of a mold. An AXL-600AW YAG laser was employed in this study. Multi-tracks were carried out to fabricate the test specimens. The optimized process parameters for laser cladding and laser re-melting experiment were presented in Table 2. The optimized process parameters were obtained according to our preliminary studies on the effects of process parameters on the morphology and mechanical properties of the WC/Fe-based composite coatings which will be published later. During the laser cladding process, the molten pool was shielded by inert gas (Ar) with a flow rate of 15 L/min.

Experiments of the effects of different heat treatment processes on laser coatings were carried out. Preheating and annealing of the specimens were performed in a heating platform and furnace, respectively. Substrate with preplaced mixed powders was preheated to different temperatures and then laser cladded. During the laser cladding process, the substrate was heated continuously to maintain a stationary temperature. After laser cladding, the specimens with coatings were annealed at 300 °C for 1 h.

### 2.3. Microstructure and Properties

The geometrical profiles and the cracks of the composite coatings were analyzed by XTL2400 stereo microscope. Microstructure was analyzed by OLYMPUS-GX51 optical microscopy (OM) and Phenom proX scanning electron microscopy (SEM) equipped with energy dispersive spectrometer (EDS). Hardness was measured in a HVS-1000Z microhardness tester with a load of 1.96 N and a dwelling time of 10 s. Transverse rupture strength (TRS) of the specimens was measured by a three-point bending test with WD-300K universal testing machine. The size of the specimens was 40 mm × 4 mm × 3 mm. Because the thickness of the coating after being ground flat is small, the specimen for bending test is composed of a coating with a thickness of 0.4 mm on a medium carbon steel substrate. The resulting fracture surfaces from the bending test specimens were examined by SEM for evaluating the fracture characterization.

To evaluate the wear resistance of the crack-free WC/Fe-based composite coating, dry sliding wear tests were performed on an MPX-2000 pin-on disc wear tester. The rotational speed of the disc (Ф34 × 6 mm) was 370 r/min and the load was 500 N. The pin (Ф10 × 20 mm) was hardened steel with 0.45% C (AISI 1045), whose hardness was 53.5 HRC. The wear mass loss was measured at intervals of 15 min by electronic balance with an accuracy of 10^−4^ g. The surface of the laser cladding coating was ground by grinding wheel before the wear test. The counter wear resistance of the WC/Fe-based composite coating and the hardened steel was determined by using the wear mass loss method with an average of five repeated tests. The wear damage behaviors of the laser cladding coating were examined by SEM.

## 3. Results and Discussion

### 3.1. Influence of Laser Re-Melting

#### 3.1.1. Macro-Profile and Cracking of Coatings

**Figure 1:**
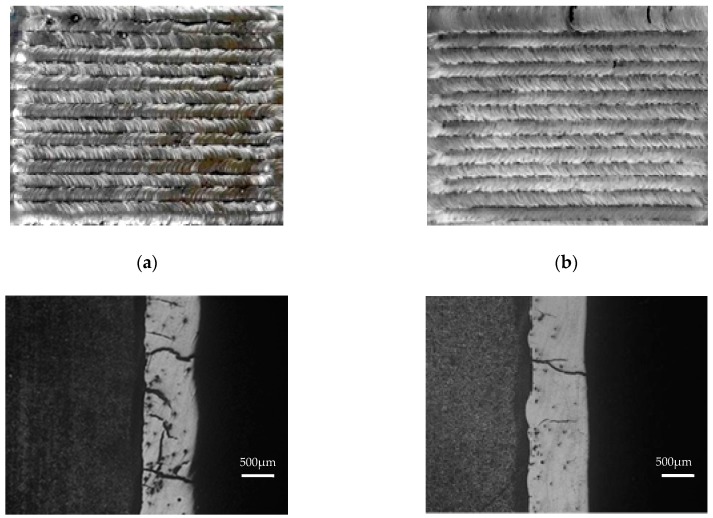
Morphology of the coatings before and after laser re-melting.

Figure 1 shows the geometrical morphology of WC-reinforced Fe-based composite coatings before and after laser re-melting. As presented in Figure 1a, a wavy appearance can be seen on the surface of the cladding coating. Serious macro-cracks, pores, and non-melted particles are also clearly seen. After laser re-melting, the surface of the cladding coating becomes smooth, as shown in Figure 1b. Pores and non-melted particles are also rarely found. Cracks are fewer and smaller. The difference of the cracks of the coating before and after the re-melting process can be further seen in Figure 1c,d, which is the morphology of the cross section of the coating. In addition, it is known that the deviation of the surface of the cladding coating is reduced by 52.8% after re-melting process. Moreover, the machining allowance will be much smaller in the subsequent finishing process in order to achieve the desired geometry, assembling tolerance, and surface integrity.

It is known that the wavy appearance on the laser coating is due to the overlapped laser scans during laser cladding process. During the re-melting process the cladding coating is re-melted to form a molten pool, and the non-melted particles in the coating can be melted into the metal pool. The wave trough will be filled by the molten metal. Thus, the difference in height between a crest and a trough reduces. In addition, during re-melting process the temperature of the laser coating and substrate is much higher compared to the laser cladding process. As a result, a lower cooling rate and temperature gradient is obtained. The thermal stress will be smaller, which is beneficial to lower cracking susceptibility.

#### 3.1.2. Microstructure and Hardness

**Figure 2:**
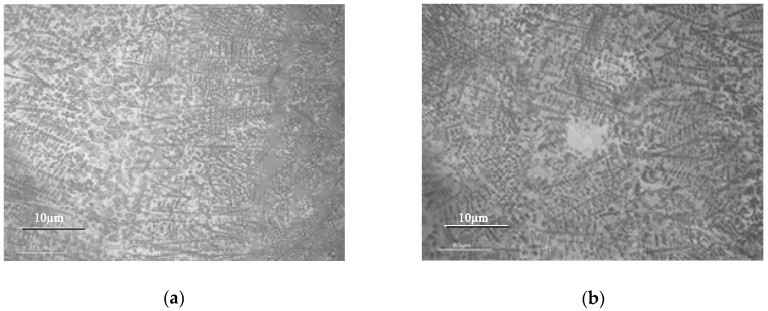
Microstructures of the coatings before and after laser re-melting. (**a**) cladding coating of WC/Fe, (**b**) re-melting of WC/Fe.

Figure 2 shows the microstructures of the laser cladding coating and the re-melting coating near the coating surface. It can be seen from Figure 2a,b that the microstructures of the cladding coatings are characterized by coarse columnar dendrites. Some WC particles disperse independently in the coating. The microstructure of the re-melting coating is a little coarser and less oriented. The microstructure characteristics are related to the ratio of temperature gradient G to solidification rate R, i.e., G/R [3]. As mentioned above, smaller cooling rate and temperature gradient are obtained during re-melting process. Also, the heat removal of the molten pool is non-directional. Therefore, it is easier to form equiaxed dendrites in the re-melting coating.

**Figure 3:**
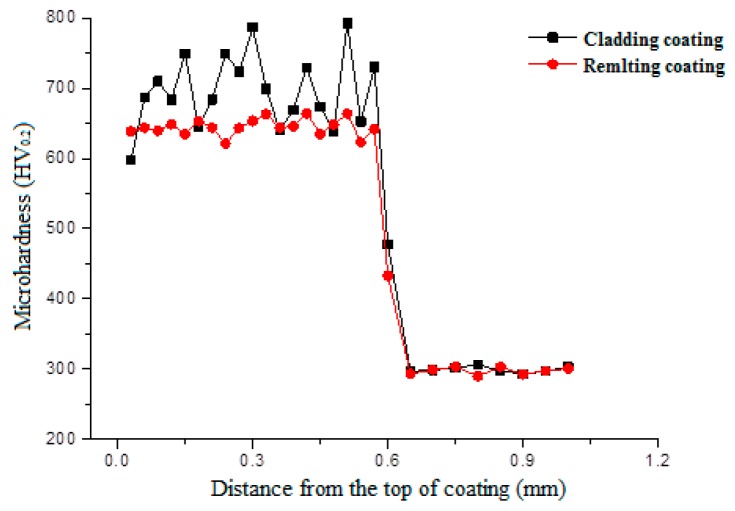
Microhardness distribution of coatings.

Figure 3 shows microhardness profiles of the cladding coating and re-melting coating. It can be seen that the microhardness of both coatings is several times higher than that of the substrate (290.5 HV0.2). The hardness distribution in the re-melting coating is much more uniform than that in the cladding coating. However, the average microhardness of the re-melting coating is 644.5 HV0.2, which is a little smaller than the average microhardness of the cladding coating (696.7 HV0.2). This is because lower cooling rate and temperature gradient are obtained during the re-melting process, which leads to smaller fine-grain strengthening in the re-melting coating. Additionally, the chemical composition and microstructure of the re-melting coating are more uniform due to the melting and diffusion of the elements and the decomposing and re-precipitating of some WC particles during the re-melting process.

### 3.2. Influence of Heat-Treament

#### 3.2.1. Crack of Coatings

**Figure 4:**
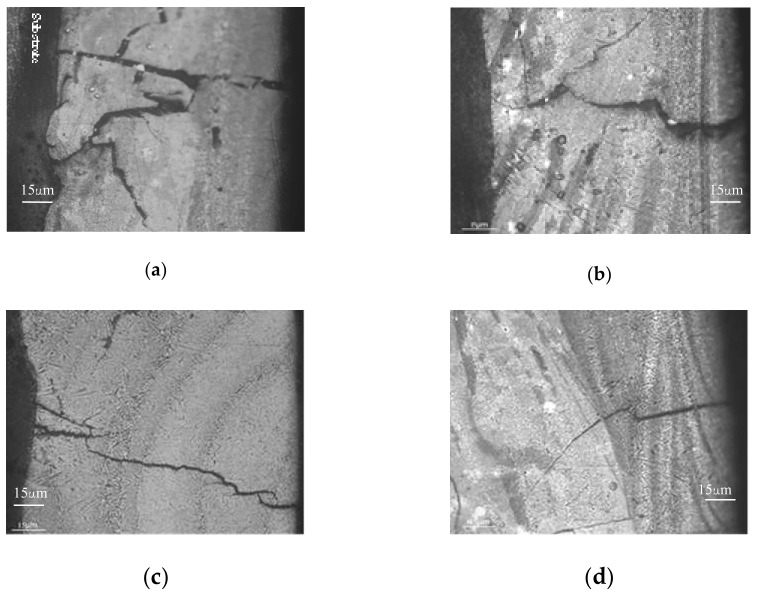
Crack characteristics in the coatings by different heat treatments. (**a**) coating 1, (**b**) coating 2, (**c**) coating 3, (**d**) coating 4.

In this section, the coatings with dimensions of 25 mm × 15 mm × 0.8 mm fabricated by laser re-melting were heat-treated with different parameters. The influence of heat treatment parameters on the number of cracks in the coatings is presented in Table 3. The calculation was taken in an area of 10 mm × 15 mm. There was a mass of cracks at the surface of the coatings without preheating the substrate or when the preheating temperature was low. The cracks were wide and crossed. However, wide cracks are basically perpendicular to the coating–substrate interface, which usually propagates throughout the whole coating, as shown in Figure 4a,b. When the preheating temperature increases to 250 °C, the number of cracks decreases significantly, and the cracks are smaller as shown in Figure 4c,d.

It can be seen from the above results that preheating the substrate and keeping it at a stationary temperature during the laser cladding process are effective crack restraining methods, while the annealing of the coating exhibits no obvious effects on the formation of the cracks. This is because thermal stress is released due to the reduction of the temperature gradient and the smaller cooling rate by preheating the substrate [12,17]. Otherwise, a mass of cracks is formed owing to the big temperature gradient and cooling rate. When cracks are formed in the coatings, the annealing of the coating cannot eliminate the formed cracks but can further relieve the residual stress.

#### 3.2.2. Hardness and Microstructure

**Figure 5:**
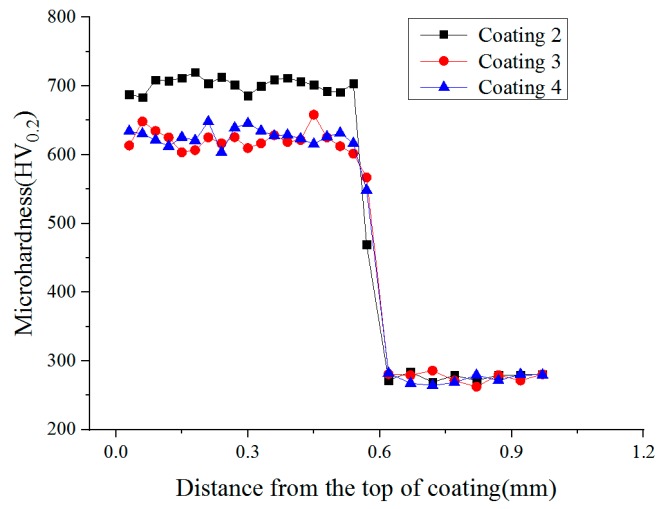
Microhardness distribution of coatings prepared with different heat treatments.

**Figure 6:**
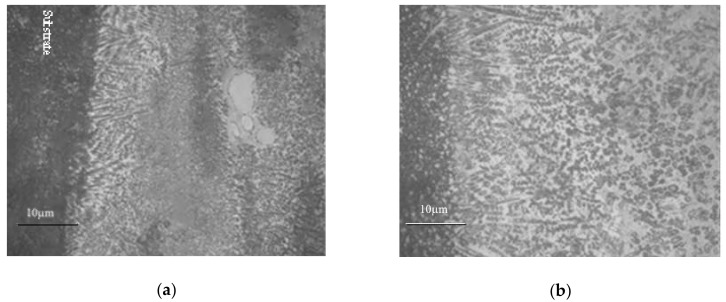
Microstructures of coating 2 and coating 3. (**a**) Coating 2 (annealing at 300 °C for 1 h), (**b**) coating 3 (preheated to 250 °C).

WC-reinforced Fe-based composite coatings are intended for wear resistance applications. Consequently, the application of heat-treatment on the coatings should be done without deteriorating their high hardness. Therefore, the preheating temperature cannot be set too high, although high preheating temperature benefits the relieving of thermal stress in the coatings. Figure 5 shows microhardness profiles of the cladding coatings heat-treated by different parameters. It reveals that the annealing of the coating has no effect on the hardness distribution in the coating but the average microhardness of the coating changes from 644.5 HV0.2 to 621.8 HV0.2 when the substrate is preheated to 250 °C. This is because annealing process does not change the microstructure of the coating, whereas preheating the substrate leads to the reduction of the temperature gradient and the cooling rate. As a result, coarser grains are obtained, as shown in Figure 6a,b. It is clearly visible that coarser grains appeared in the bottom of coating 3.

### 3.3. Influence of Co Addition

#### Cracking Tendency and Microstructure

It is known from the above experimental results that crack initiation cannot be restrained completely by applying the re-melting process and preheating the substrate. As a result, crack-free WC-reinforced Fe-based composite coatings cannot be obtained for laser cladding large areas of coating. In order to solve the crack problem and to not significantly lower their hardness, different amounts of Co was added to toughen the coatings. Coatings with dimensions of 45 mm × 40 mm × 0.8 mm were fabricated. The influence of Co on the cracking tendency of the coatings was evaluated by counting the number of cracks. The results revealed that the number of cracks decreased with the increase of Co. When 5% (weight percentage) Co was added, only one crack appeared in the coating. When the amount of Co was increased to 8%, a crack-free WC/Fe coating was obtained.

**Figure 7:**
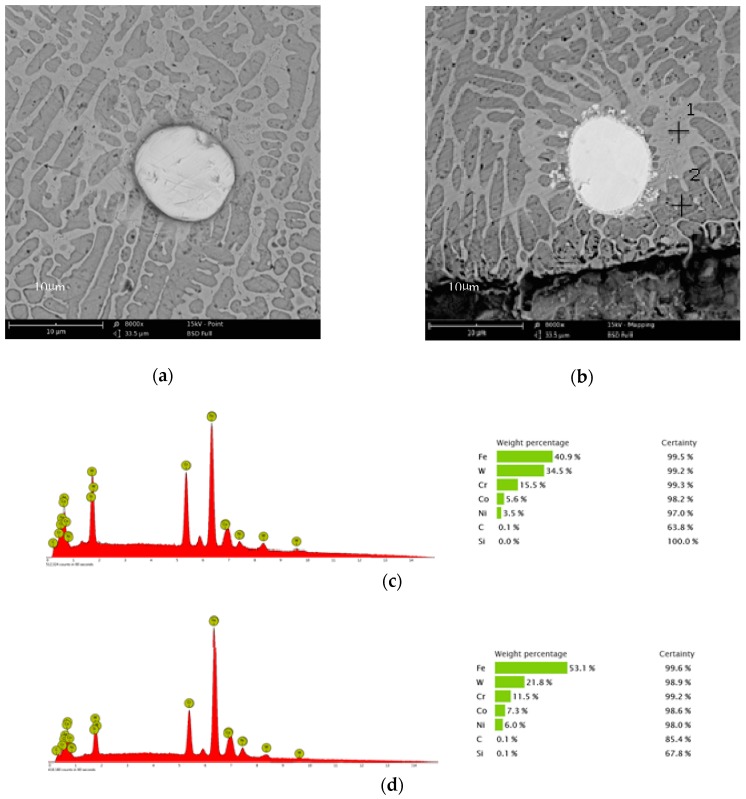
The influence of Co on the microstructures of coatings (**a**) without addition of Co, (**b**) with 8% Co addition. (**c**) EDS of point 1, (**d**) EDS of point 2.

Figure 7 compares the microstructures of the coating without addition of Co and the coating with 8% Co addition. By comparing Figure 7a,b, it can be seen that the phase constituents and phase morphologies are not obviously different between the two coatings. However, there is visible difference at the interface between the WC particles and the base metal. As seen in Figure 7b, quite a number of small white particles distribute around the interface between the WC particles and the base metal in the coating with 8% Co addition. The bonding between WC particles and the base metal is better in the coating with 8% Co addition than in that without addition of Co. EDS spot analysis of the constituent phase near WC particles in Figure 7b is presented in Figure 7c,d.

**Figure 8:**
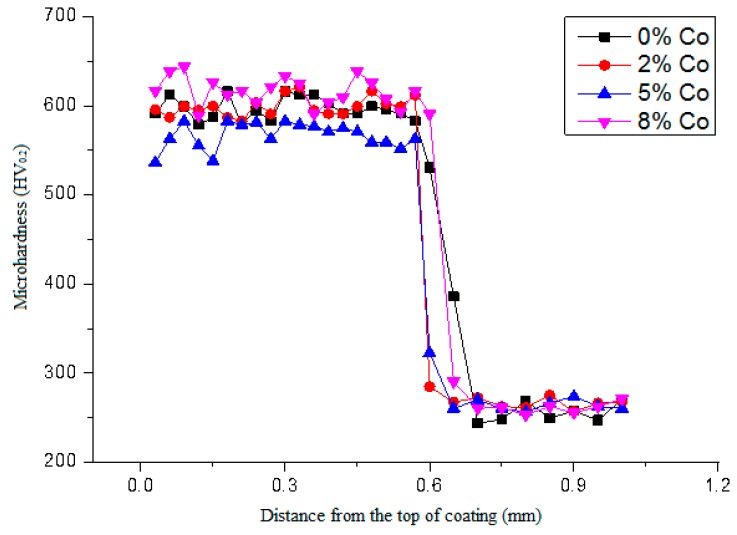
The influence of Co on the microhardness

Figure 8 shows the microhardness profiles of the cladding coatings with different amounts of Co addition. It can be noted that the influence of Co on the microhardness of the coatings fluctuates in a small range but remains high overall.

**Figure 9:**
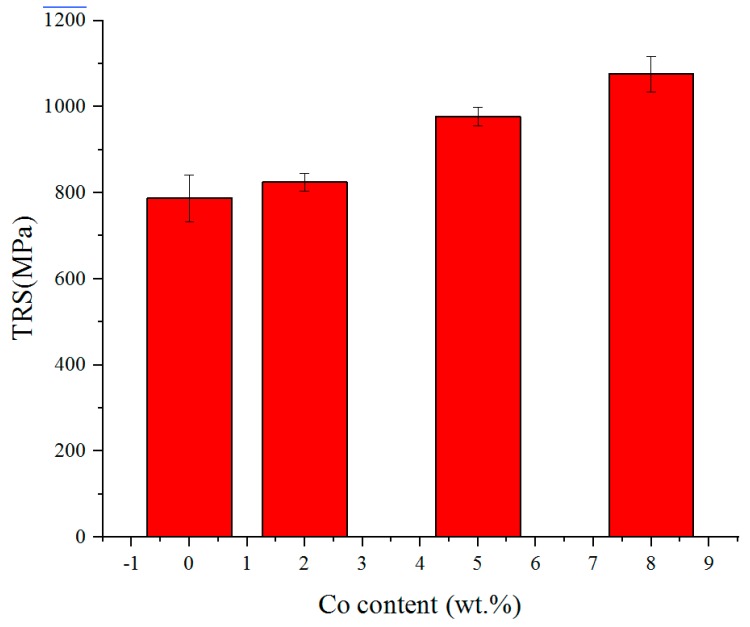
The influence of Co on transverse rupture strength (TRS).

**Figure 10:**
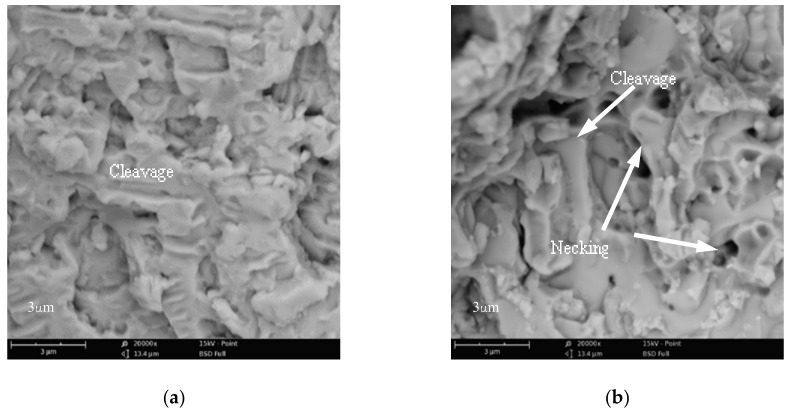
SEM observation of the fracture surfaces of WC/Fe composite coatings (**a**) without Co and (**b**) with 8% Co.

The influence of Co on the transverse rupture strength (TRS) of the coatings is presented in Figure 9. The TRS of the coatings increases with the increase of Co addition. The results reveal that addition of Co can toughen the WC/Fe composite coating. Consequently, the crack susceptibility is reduced, as demonstrated above. Morphology of fracture surfaces of the bending test samples provided further insight into the effects of Co addition on the fracture characterization of the WC/Fe composite coating. As shown in Figure 10b, the cleavage fracture and ductile fracture with necking are noticeable, which indicates that the coating with 8% Co exhibits a combination of ductile rupture as well as brittle fracture. However, in Figure 10a, only cleavage fracturing is seen, implying that the coating without Co addition is brittle. The characteristics of the fracture morphology are in accordance with the results of the TRS.

**Figure 11:**
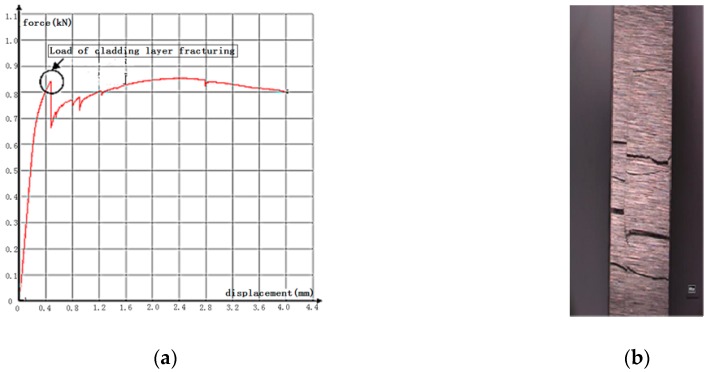
(**a**) Load–deformation curve of coating with 8% Co, (**b**) Specimen after bending test.

An interesting characteristic of the load–deformation curve of three-point bending test of the WC/Fe composite coating is noticed. As shown in Figure 11a, there are several points of abrupt change in the curve. It is found that the first point of the load decreasing abruptly corresponds to the fracture beginning in the coating. Therefore, the maximum load at this point is used to calculate the TRS of the coating. Because the specimen for the bending test is composed of the coating and the medium carbon steel substrate, when the coating begins to fracture, the medium carbon steel substrate will continue to deform due to its good plasticity. At the same time, cracks continue developing in the coating until the specimen fractures in the whole cross section, as shown in Figure 11b. Hence, it can be concluded that each point of abrupt change in the curve implies a new fracture forming in the coating.

Although the addition of Co seems to have no obvious effects on the phase constituents and phase morphologies as well as hardness of the WC/Fe composite coating, the TRS of coatings with Co addition is increased and the crack tendency is effectively inhibited. This may contribute to the role of Co in promoting the dissolution, diffusion, and interaction of WC with Fe-based metals. Proof of this is the fact that WC is tightly attached to the base metal, and small particles precipitated near the interface of the WC and base metal, as shown in Figure 7b. It has been reported by previous researchers that during the laser cladding process, WC particles easily suffer from dissolution in the molten Fe-based alloy. The dissolution of tungsten carbide results in significant presence of tungsten in the matrix, even with the formation of W2C and WC phases [1,3,18]. The dissolution of tungsten carbide and the diffusion of tungsten in the base metal can be further confirmed according to the results of the above EDS analysis. As shown in Figure 7c,d, the constituent phases near the interface of the WC and base metal are rich in W.

### 3.4. Wear Resistance of the Coating

**Figure 12:**
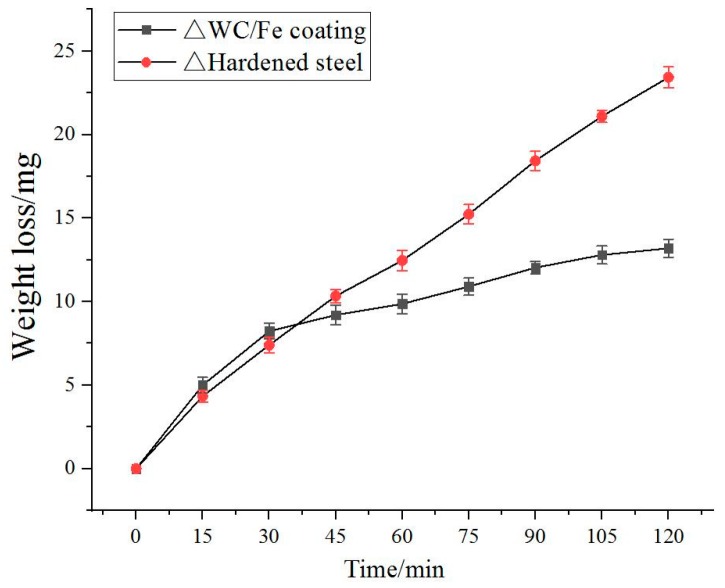
Wear mass loss of the counter bodies against wear test time.

The variations of the wear mass loss of the counter bodies of the above crack-free WC/Fe composite coating and the hardened medium steel with 0.45% C (AISI 1045) with the wear test time are shown in Figure 12. The results show that the wear mass loss of the WC/Fe composite coating is similar to that of the hardened medium steel at the beginning of the wear test. However, the wear mass loss of the WC/Fe composite coating is lower than that of the hardened medium steel when the wear test time exceeds 30 min, and the difference between them increases with time. It can be seen that the wear resistance of the WC/Fe composite coating is enhanced by 1.8 times compared with the hardened medium steel according to the average wear mass loss when the wear test lasts 120 min.

**Figure 13:**
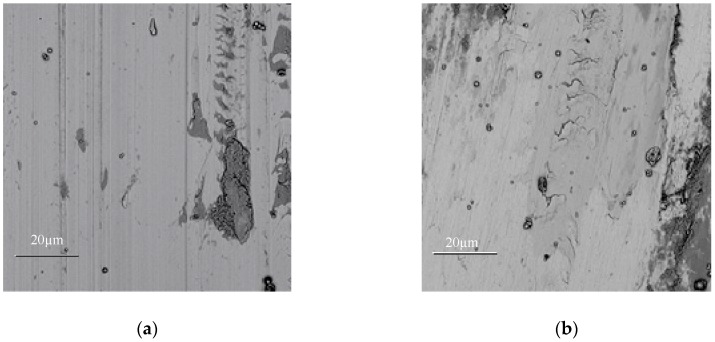
Morphologies of the counter bodies after 2 h wear testing. (**a**) Hardened steel, (**b**) WC/Fe coating.

As demonstrated above, the average microhardness of the crack-free WC/Fe composite coating (621.8 HV0.2) is a little higher than that of the hardened medium steel (about 570 HV0.2). However, in the WC/Fe composite coating, the WC reinforcement particles are wrapped by the Fe-based metal whose hardness is low. At the beginning of the wear test, the wear mass loss of the WC/Fe composite coating is mainly loss of Fe-based metal. After that, the WC reinforcement particles are exposed to the base metal. Therefore, the wear mass loss of the WC/Fe composite coating is lower than that of the hardened steel. The SEM morphologies of the counter bodies of the WC/Fe composite coating and the hardened steel after 120 min wear testing are presented in Figure 13. As shown in Figure 13a, the hardened medium steel exhibits deep grooves and black patches. By comparison, the wear surface of the WC/Fe composite coating is smoother (13b). The smoother wear surface is attributed to the higher microhardness of the cladding layer.

## 5. Conclusions

The laser re-melting process is helpful to reduce the cracking susceptibility and other defects of coatings. Also, surface deviation reduced significantly and the hardness of the coating distributed more uniformly but decreased slightly. Many wide and crossed cracks were found at the surface of the coatings when the cladding was performed on the substrate without being preheated or with low preheating temperature. Cracks in the coating can be inhibited effectively by preheating the substrate to 250 °C and maintaining the temperature during the laser cladding process, as well as applying an annealing treatment at 300 °C for 1 h. With these heat treatment conditions, the cladding coating retains its high hardness. Crack initiation cannot be restrained completely by applying the re-melting process and heat treatment of the coating. Crack-free WC/Fe coatings can be manufactured when 8% Co is added. This could contribute to the role of Co in promoting the dissolution, diffusion, and interaction of WC with the Fe-based metals. The wear resistance of the crack-free WC/Fe coating is much better than that of the hardened medium steel, especially when the wear time is long.

## Figures and Tables

**Table 1:** Chemical composition of the Fe-based alloy (wt.%).

Cr	Ni	B	Si	C	Fe
14.8~15.2	9.2~11.1	0.9~1.1	0.9~1.1	0.09~0.1	Bal.

**Table 2:** Processing parameters for laser cladding and laser re-melting.

Voltage (V)	Electric Current (A)	Fluency (Hz)	Scanning Speed (mm/s)	Pulse Width (ms)	Beam Diameter (mm)	Track Distance (mm)
380	120	30	360	3	1.5	1

**Table 3:** Influence of heat treatment parameters on cracks.

Heat Treatment Method/Number of Cracks	Coating 1	Coating 2	Coating 3	Coating 4	Coating 5	Coating 6	Coating 7
Preheating (°C)	—	—	250	250	50	150	150
Annealing (°C)	—	300	—	300	—	—	300
Cracks	14	15	4	4	14	10	10
